# Athothis, a Satire on Modern Medicine

**Published:** 1887-09

**Authors:** 


ATHOTHIS. A Satire on Modern Medicine. By Thomas C. Minor. Cincinnati: Robert Clarke & Co. 1887.

Apt, attractive and well-written, will repay one for the time spent in its perusal, the keen thrusts at medical follies and weaknesses affording amusement, while beneath the rippling surface of sarcasm lie pools of truth that thought and reflection will do well to fathom. But to read this book without gaining a lesson for the future is time wasted. "Vanity of vanities" is a good text, but a poor motto. Blues and biliousness go hand-in-hand, and blue-pill is good for both of them. When in health, man is a cheerful being. The right course is to make an insight into our faults, but an inspiration to better conduct and higher achievements; to

——"rise on stepping-stones
Of our dead selves to higher things."

